# Activation of the Complement System in the Lower Genital Tract During Pregnancy and Delivery

**DOI:** 10.3389/fimmu.2020.563073

**Published:** 2021-01-11

**Authors:** Sivan Livson, Hanna Jarva, Ilkka Kalliala, A. Inkeri Lokki, Jenni Heikkinen-Eloranta, Pekka Nieminen, Seppo Meri

**Affiliations:** ^1^Department of Obstetrics and Gynecology, Helsinki University Central Hospital, University of Helsinki, Helsinki, Finland; ^2^Department of Bacteriology and Immunology and Translational Immunology Research Program, University of Helsinki, Helsinki, Finland; ^3^HUS Diagnostic Center, Helsinki University Hospital Laboratory, Helsinki University Hospital, Helsinki, Finland; ^4^Department of Surgery and Cancer, Institute of Reproductive and Developmental Biology, Imperial College London, London, United Kingdom; ^5^Department of Metabolism, Digestion and Reproduction, Faculty of Medicine, Imperial College London, London, United Kingdom; ^6^Human Microbiome Research Program, Faculty of Medicine, University of Helsinki, Helsinki, Finland

**Keywords:** uterine cervix, vaginal mucosa, IgG, IgA, C3, parturition, inflammation, delivery

## Abstract

**Background:**

Human pregnancy alters profoundly the immune system. The local involvement and mechanisms of activation of the complement system in the cervicovaginal milieu during pregnancy and delivery remain unexplored.

**Objectives:**

To determine whether normal pregnancy and delivery are associated with local activation of complement or changes in the immunoglobulin profile in the cervix.

**Study Design:**

This study was designed to assess IgA, IgG, and complement activation in the cervicovaginal area in three groups of patients: i) 49 pregnant women (week 41+3–42+0) not in active labor, ii) 24 women in active labor (38+4–42+2), and iii) a control group of nonpregnant women (n=23) at child-bearing age. We collected mucosal samples from the lateral fornix of the vagina and external cervix during routine visits and delivery. The Western blot technique was used to detect complement C3 and its activation products. For semiquantitative analysis, the bands of the electrophoresed proteins in gels were digitized on a flatbed photo scanner and analyzed. IgA and IgG were analyzed by Western blotting and quantified by ELISA. One-way ANOVA and Tukey’s Multiple Comparison tests were used for statistical comparisons.

**Results:**

A higher abundance but lower activation level of C3 in both the external cervix (P<0.001) and lateral fornix of the vagina (P<0.001) was observed during delivery (58 ± 22, n= 24) in comparison to the groups of nonpregnant (72 ± 13%; mean ± SD, n=23) and pregnant women (78 ± 22%, n=49). Complement activating IgG was detected in higher abundance than IgA in the cervicovaginal secretions of pregnant women. In a small proportion samples also C3-IgG complexes were detected.

**Conclusions:**

Our results reveal an unexpectedly strong activation of the complement system and the presence IgG immunoglobulins in the cervicovaginal area during pregnancy, active labor, and among nonpregnant women. In contrast to the higher amounts of C3 in the cervicovaginal secretions during labor, its activation level was lower. Complement activating IgG was detected in higher concentrations than IgA in the mucosal secretions during pregnancy and labor. Taken together our results imply the presence a locally operating humoral immune system in the cervicovaginal mucosa.

## Introduction

A unique phenomenon of successful coexistence of the maternal immune system and the semi-allograft fetoplacental unit is seen in pregnant women (1–3). Growing knowledge about the immunological microenvironment within the female genital tract has increased the interest in understanding the immunological processes and their role in parturition (4–6). Reports describing chemotactic recruitment and activation of inflammatory neutrophils and macrophages into the uterus, decidua, fetal membranes and cervix during labor point towards local rather than systemic inflammatory events (7–10). The innate immune system has been linked to these processes and to the generation of a sterile pro-inflammatory state that will pave the way to labor and delivery of the baby (11, 12).

The complement (C) system is part of innate immunity (13, 14). The proteolytic activation cascades of C comprise about 50 proteins (15). Complement can become activated through three distinct pathways: the classical, lectin and alternative pathways (16). These pathways each converge at the central step of the C system, i.e. the cleavage of C3 by C3 convertases (16). Complement functions in antimicrobial defense and as an opsonophagocytic clean-up system of the body together with phagocytes. Activation products generated include e.g. the anaphylatoxins C3a and C5a that can induce major physiological changes, like contracting smooth muscle and increasing vascular permeability (17). Pregnancy and parturition are associated with increased levels of C components and with C activation in the blood (18–26). The presence of C components in tissues is a result of their diffusion from blood plasma and local production by different cell types including macrophages, fibroblasts, and endothelial cells. Information about the local role of C in the cervicovaginal area in humans is scarce.

Under normal circumstances C activation is well regulated and only minimal deposition of its activated components, including C1q, C4b, C3b/iC3b, or the membrane attack complex (MAC), occurs in the mucosa. However, in a variety of adverse pregnancy outcomes dysregulation of C has been demonstrated. These include hypertensive diseases of pregnancy (27, 28), antiphospholipid antibody syndrome–associated fetal loss (29), recurrent miscarriage and preterm birth (30–33). To our knowledge, local activation of the C system in the cervicovaginal mucosa in humans and its relation to timing of parturition have not yet been examined.

We hypothesized that, because of e.g. a strong microbial exposure in the lower genital tract, the C system is constantly active, but should be tightly regulated at the time of parturition in order to protect the mother and fetus from an immune attack. Our present study aimed at investigating whether and to what extent C is activated in the cervicovaginal area, and whether local C3 activation is related to the parturition process.

## Materials and Methods

### Study Subjects and Samples

To address the role of complement in parturition, a cohort of samples was collected to determine C activation in the cervicovaginal area. We recruited three groups of study subjects: i) pregnant women (n=49) with pregnancy duration of 41+3 to 42+0 weeks in whom labor had not yet become initiated, ii) women in active labor (n=24) (38+4 - 42+2 weeks), and iii) non-pregnant women (n=23) in child-bearing age, who had arrived for routine out-patient visits in the clinic. All pregnancies were singleton pregnancies with intact fetal membranes at the time of sampling. None of the women had been previously treated for cervical precancerous lesion. All women were generally healthy with no chronic disease or diagnosed immune deficiency, except for one with IgA deficiency in the pregnant women group. None of the study subjects had used any type of corticosteroids for at least 6 months before sampling. None of the women recruited had had unprotected sexual intercourse for at least 48 h before sampling. All pregnant individuals were screened for gestational diabetes in mid-pregnancy. Altogether 17% tested positive. They were treated conservatively and monitored at the maternity clinic with no need for antidiabetic drugs. Demographic and clinical characteristics of the pregnant study subjects are presented in **Table 1**.

**Table 1:** Patient characteristics.

	Non pregnant	Pregnant patients	Patients in labor
	n = 23	n = 49	n = 24
Age (mean, SD)	30 ± 5	32.3 ± 4.3	30.2 ± 5
First delivery (%)		53	33
Median prepregnancy BMI		23	22
Smoking (%)	30	4.1	4.2
Antibiotic consumption in the past 6 months (%)		22	21
Pregnancy length in days (median)		294	282
Range of pregnancy length (days)		289-296	270-92
Average baby weight (g)		3700	3574
Positive for vaginal group B streptococcus (%)		33	35
Artificially induced delivery (%)		61	0
Gestational diabetes (%)		15	18

All subjects (n=96) were in child-bearing age (17–40 years old). The median BMI value before pregnancy was 23 ± 2.4 (median ± range, n=73). In the pregnancy, delivery and control groups, systemic or local vaginal usage of antibiotics less than 6 months from sampling was recorded in 22%, 21%, and 20% of the women, respectively (**Table 1**). In the pregnancy group 53% (26/49) and the labor group 33% (8/24) of the women were primiparas (p<0.05).

All samples were collected at the Helsinki University Hospital between October 2015 and March 2017. The study was approved by the Helsinki University Hospital’s Ethical Committee (91/13/03/03/2015). All participating women provided written informed consents and were requested to fill up an exploratory questionnaire. Serial samples were collected by two experienced physicians from the lateral fornix of the vagina (LF) and external cervix (EC) using the Rovers Viba-Brush tool (Rovers Medical Devices, Oss, The Netherlands). Ten subjects provided two additional swabs from the EC and LF directly into 10 mM EDTA to control for possible *ex vivo* C activation. The additional group of 23 non-pregnant subjects were sampled from the LF. These samples were taken at a colposcopy clinic. Many of the patients came because they may have cervical changes. Thus, samples from the external cervix would not have been representative. All samples were inserted immediately into Eppendorf tubes containing 20 µl phosphate-buffered saline, pH 7.4 (PBS) and were frozen into -80°C within 30 min of sampling. No blood-contaminated samples were included in the study.

### Analysis of IgG and IgA

IgG and IgA in cervicovaginal samples were analyzed by immunoblotting and quantified by an ELISA assay. Twenty µl portions of appropriately diluted samples were loaded onto 4%–12% SDS-PAGE gels under reducing conditions. After transferring the proteins to a nitrocellulose filter, nonspecific binding sites were blocked by incubation with 5% milk in phosphate-buffered saline (PBS) containing 0.05% Tween 20 detergent. The membranes were then incubated for 1 h at RT with HRP-conjugated rabbit-anti-human IgG or IgA antibody (Dako; final dilutions 1:10,000 and 1:5,000, respectively, in milk/PBS/Tween). Protein bands were visualized by an in-house protocol for electrochemiluminescence. After washing with PBS+0.05% Tween 20 an enhanced chemiluminescence solution (WesternBright ECL, Advansta, San Jose, CA) that includes hydrogen peroxide was added and films developed at different exposure times.

To quantify levels of IgG and IgA in the cervicovaginal mucosal samples standardized ELISA assays (Bethyl Laboratories, Inc. USA, Catalog no. E101-104 and Catalog no. E100-102, respectively) were used. Human sera with known IgG and IgA concentrations were used as controls. In this assay the intra- and inter-assay coefficients of variations were less than 5%. Mucosal samples and sera were diluted 1:1000, 1:3000, and 1:10000 for IgG and 1:10, 1:30, 1:100, and 1:300 for IgA and analyzed in duplicate. After incubating the samples for 2 h on the plate wells they were washed with a buffer (TBS) containing 0.05% Tween 20 using an automated plate washer (EL*x*50 Washer, BioTek). Thereafter, HRP-anti human IgG and HRP-anti-human IgA (both from Dako), diluted 1:5000 and 1:2000 respectively in PBS were added and incubated for another 2 h at 22°C. After washing with TBS, 0.05% Tween 20, substrate (OPD) was added. The reaction was stopped with 0.5M H_2_SO_4_ and a microplate spectrophotometer (SpectraMax, Bio-strategy) was used to measure the optical density of samples at a wave-length of 492 mm.

### Analysis of C3 Cleavage in Vaginal Lateral Fornix and External Cervix Samples

Western blot analyses were performed using in-house protocols (34). The samples were thawn on ice, centrifuged 12,000 × *g* for 3 min and diluted 1:10 in sterile PBS. 20 µl portions of a dilution series of samples (in PBS) in SDS and 5% mercaptoethanol containing sample buffer were loaded onto 4%–12% SDS-PAGE gels and run under reducing conditions. A normal human serum (NHS) pool was obtained from healthy laboratory personnel after a written informed consent and used as a reference. The proteins from the gel were electrotransferred to a nitrocellulose filter. To prevent nonspecific binding the nitrocellulose membranes were incubated in 5% milk in PBS/Tween 0.05% for 1 h. The membranes were then incubated with rabbit anti-human C3c antibody (Dako; final dilution: 1:10,000 in milk/PBS/Tween) overnight at +4°C. For additional detection of C3-IgG complexes, also rabbit antibodies against C3d (Dako) were used similarly as anti-C3c antibodies. The membranes were washed with PBS/Tween and incubated for 1 h at RT with HRP-goat-anti-rabbit IgG antibody (Jackson ImmunoResearch; 1:10,000 in milk/PBS/Tween). Finally, the membranes were washed, and protein bands were visualized by electrochemiluminescence.

### Quantification of C3 Activation

For quantitative determination of the C3 bands the films were digitized on a flatbed photo scanner and quantified using ImageJ/Fiji win-64 software. Activation of C3 results in several split products (**Figure 2**), including C3b, iC3b, C3c, C3dg, and C3d. The level of C3 activation was determined by assessment of the relative level of intensity of the C3α-chain split products (α'-chain fragments in the split products) from total C3 reactivity. The respective α'-chains of these split products can be visualized by Western blotting and may be quantified in relation to the amount of total C3 α/α'-chain reactivity (native plus activated C3) found in the same specimen. The β chain is not cleaved, thus its amount remains the same regardless of the level of activation. Calculation of the total percentage of C3 activation was done by calculating the intensity of C3α' split products [x 100%] and dividing the result by [intensity of native C3α + C3α' split products].

Results are shown as mean ± SD values. For comparing the significances of differences between the study groups the two-tailed Student’s t-test or one-way ANOVA with Tukey´s multiple comparison test were used.

## Results

### Immunoglobulins IgA and IgG in the Cervicovaginal Samples

**Figure 1:**
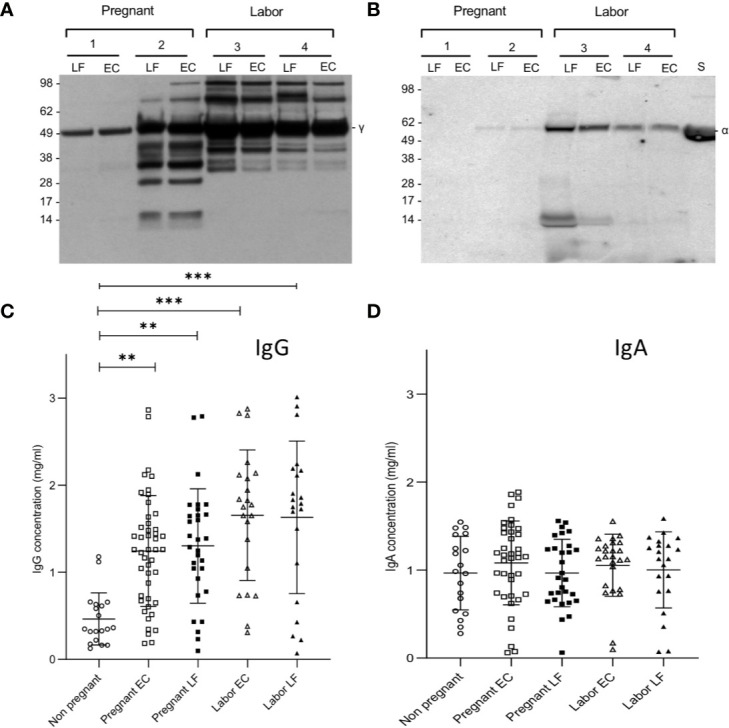
Immunoblotting analysis of IgG and IgA heavy chains and levels of IgG and IgA in EC and LF during pregnancy and labor. Samples 1 and 2 were from pregnant women and samples 3 and 4 from women in labor. As shown in **(A)**, IgG is abundant in all samples. More quantitative variation exists for IgA **(B)**. In the selected samples from pregnant women patient number 1 was found to be IgA-deficient. Antibodies used were specific for the γ (IgG) and α (IgA) heavy chains, both approximately 50 kDa in size. Quantification of IgG **(C)** and IgA **(D)** was done by ELISA. Higher concentrations of IgG were found in the EC and LF during pregnancy and labor in comparison to IgA. IgG concentration is significantly higher during pregnancy and during labor both in the EC and the LF in comparison to the nonpregnant controls (**P < 0.01, ***P < 0.001; one-way ANOVA and Tukey´s multiple comparison test).

Since IgG is known to activate complement, but IgA is not, we wanted to analyze the relative proportions of these immunoglobulins in the cervical secretions. **Figures 1A, B** show two representative Western blots for analysis of the IgG and IgA heavy chains, γ and α, respectively. In the majority of samples (n=96) the IgA heavy chains were intact. For IgG, however, in approximately half of the samples, especially in those taken during delivery, additional bands with a lower molecular weight than that for the intact 50 kDa heavy chain were observed in the blots. This suggests proteolytic cleavage. The intensities of the IgG heavy chains were stronger than those for IgA suggesting that IgG is abundantly present in all the mucosal samples. In one case an apparent IgA deficiency was detected (**Figure 1B**).

More accurate quantification of IgG and IgA was done by ELISA (**Figures 1C, D**). On the average, higher concentrations of IgG than IgA were observed in samples from pregnant women (both in those in late pregnancy or in labor; P<0.001: one-way ANOVA and Tukey´s multiple comparison test). In contrast, in nonpregnant women the IgA levels (1.0±0.4, n=19; mean ± SD) were higher than IgG levels (0.5±0.3, n=19, P<0.001, Student’s t-test). The IgG levels were higher in samples taken during labor (EC: 1.7±0.7, n= 22; LF: 1.6±0.9, n=21) or late pregnancy (EC: 1.2 ± 0.6 mg/ml, n=46; LF: 1.3±0.7, n= 30) than in those from nonpregnant women (0.5±0.3, n=19, P<0.01; Tukey´s multiple comparison test). Values during labor (both in EC and LF) were slightly higher than in late pregnancy (P<0.01). No significant difference was detected between the study groups in IgA concentrations.

### Local C3 Activation in Non-Pregnant and Pregnant Women and During Labor

**Figure 2:**
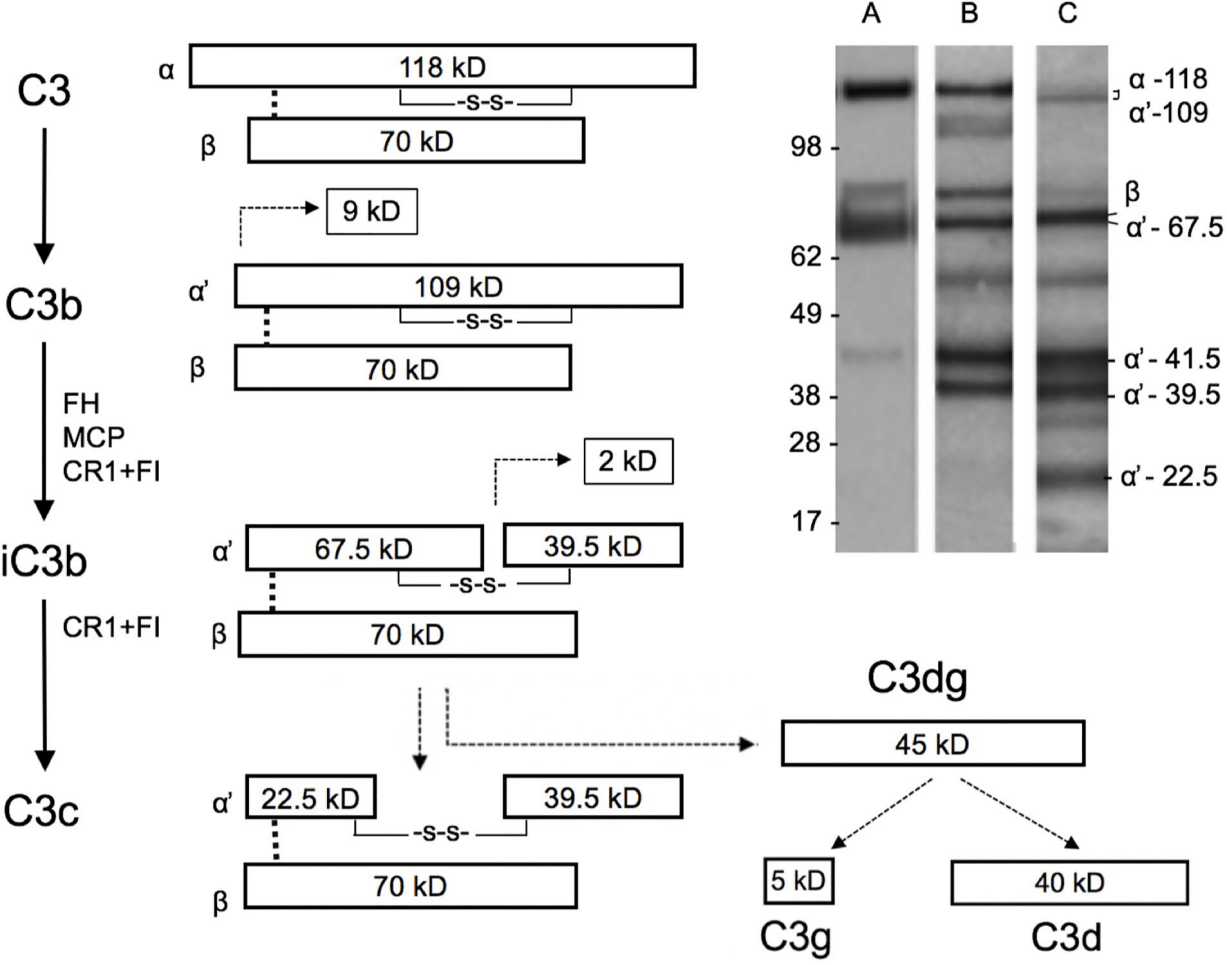
Schematic structure of C3 and its cleavage. The three distinct complement pathways will converge at C3 and promote activation of C3 to C3a and C3b by C3 convertases. The 109 kDa alpha´-chain in C3b becomes cleaved by factor I and a cofactor, e.g. factor H, to smaller 67.5 and 39.5 kDa fragments that are then part of iC3b. iC3b will subsequently get further cleaved to C3c and C3dg. The β chain remains uncleaved as a 70 kDa band. Examples of the C3 cleavage patterns (determined by rabbit anti-human C3c antibody) in our samples are shown in the upper right corner. Lanes A, B, and C show representative samples of different levels of C3 breakdown in the patient samples. Lanes A, B, and C represent samples containing C3b (A), iC3b (B), and a mixture of iC3b and C3c (C), respectively. In lane B, and faintly in lane C, the band at 118–109 kDa represents alpha/alpha-chains, which have remained uncleaved. The identity of the ≈55 kDa band is not known, but could represent a proteolytic cleavage fragment of the 67.5 kDa band. Such a fragment could be generated e.g. by plasmin, which is likely present in the mucosal fluids. The fragment above the 70 kDa beta-band could represent an autolytic cleavage product of the alpha-chain, where C3a (9kDa) has remained bound to the alpha’-67.5 kDa fragment.

**Figure 3:**
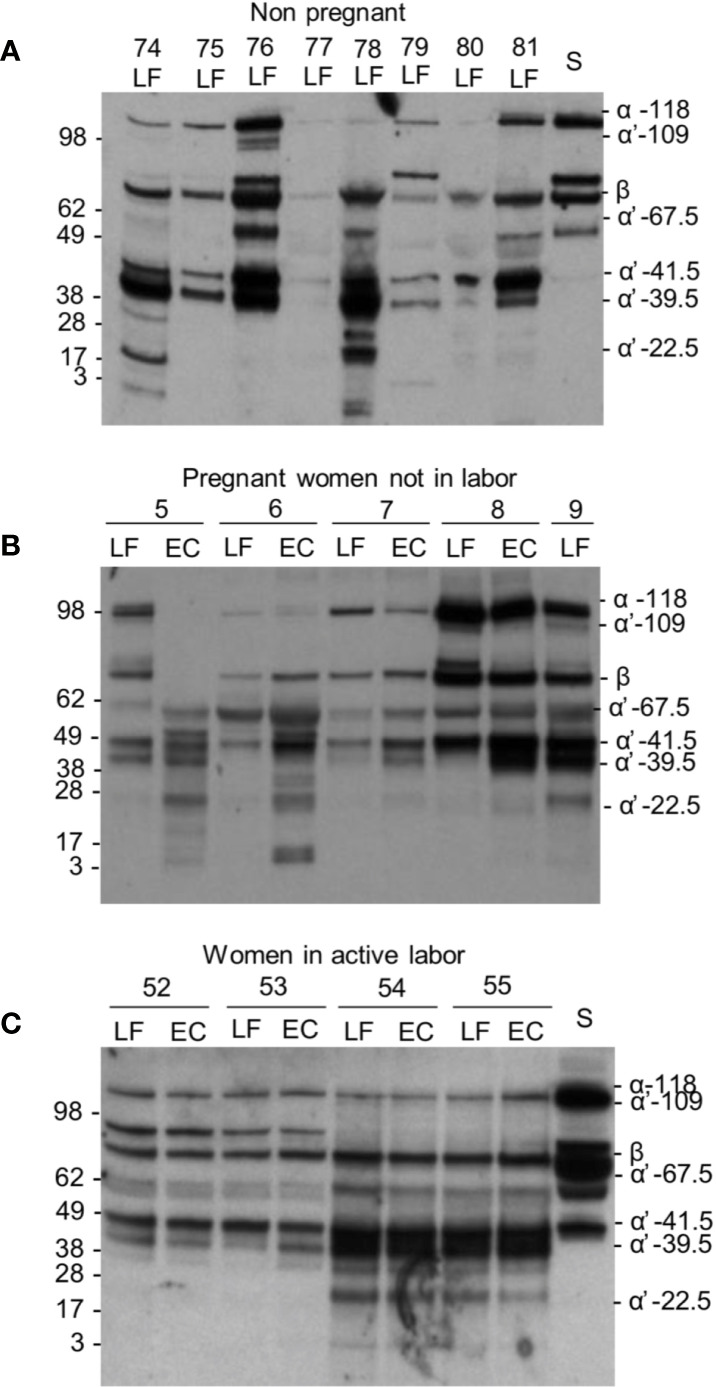
C3 cleavage in the cervicovaginal area. Examples from the three different sample groups are shown. Samples were from: **(A)** women in the nonpregnant group; **(B)** pregnant women, who are not in labor and **(C)** women in active labor but with intact membranes. The cleavage was studied by running the samples in SDS-PAGE under reducing conditions and using Western blotting with an anti-C3c antibody. Sample numbers and types within each group are indicated on the top of the blot. As shown, a wide variation in the extent of C3 cleavage occurs in the samples. The β chain and the key α´-chain fragments are indicated on the right. Molecular weight markers (kDa) are indicated on the left. S, serum control.

Our next aim was to study the presence and activation of C3 locally in the cervicovaginal area. Native complement C3 consists of one α and one β chain linked together *via* a disulfide bond. The activation of C3 is schematically described in **Figure 2**. The band identities in the immunoblots were determined by comparison to C3 activation fragments in zymosan-activated serum and by inactivating purified C3b with factors H and I (not shown). During activation, the C3 β-chain remains intact in the cleavages and thus reflects the total original amount of C3 in the sample. In addition to the α-chain the Western blots traced the α’-chain and its cleavage fragments, which were quantified by using the “ImageJ/Fiji win-64 software J”. As shown in **Figure 3**, C3 in the cervicovaginal samples becomes extensively activated and degraded into multiple cleavage fragments. Occasionally, in the blots a band above the C3 beta-chain was observed (**Figure 3**). It is likely an autolytic cleavage product of the C3 alpha-chain, and thus not an activation product.

**Figure 4:**
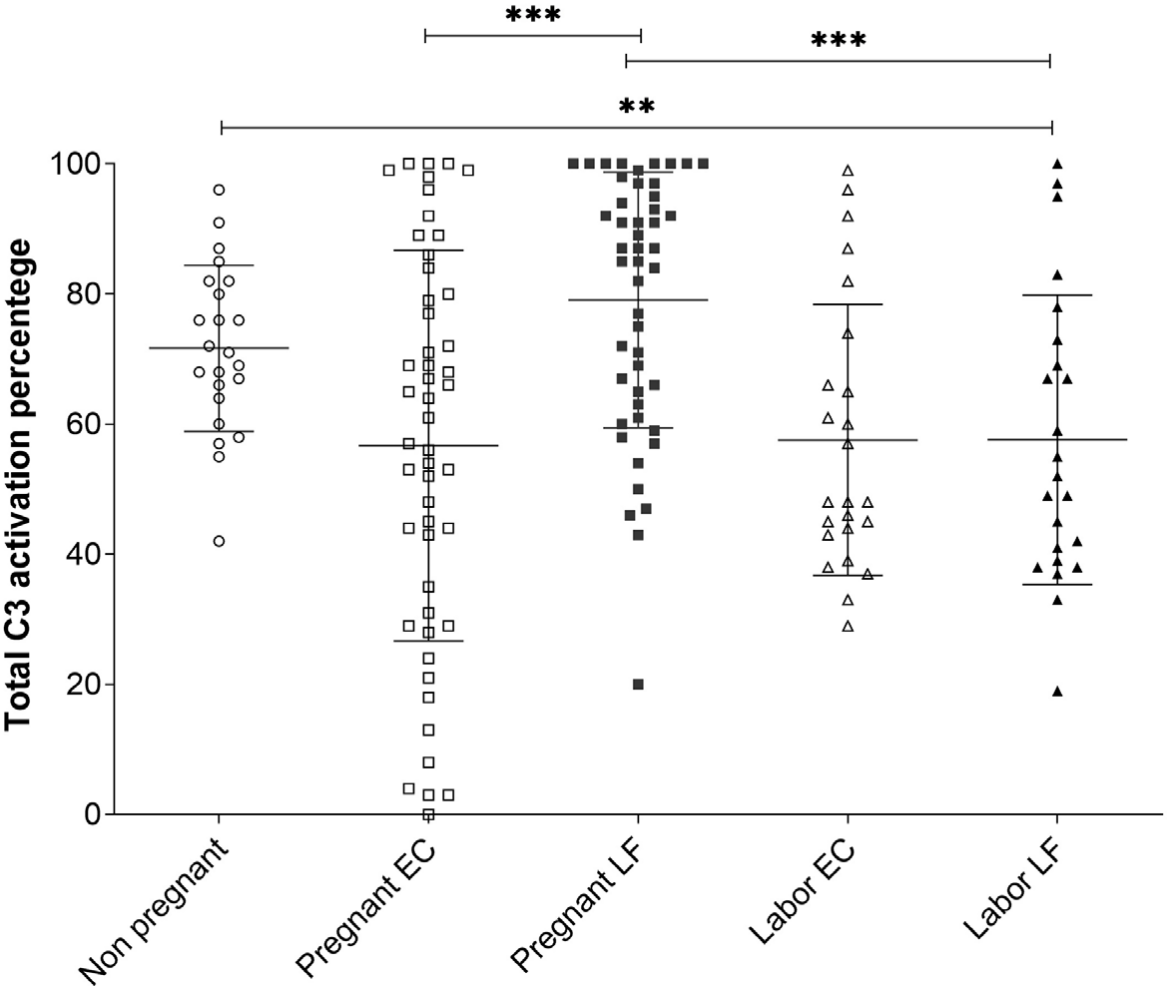
Comparison of C3 activation levels in samples from the EC and the LF in the 3 different groups. The percentages of C3 activation in the three groups were determined from the Western blot analysis of C3 split products using the “ImageJ” program. The mean ± SD values for the different groups were: nonpregnant women 72 ± 13% (n=23), pregnant women EC 57±30%, LF 79 ± 20% (n=49) and women in labor 58 ± 22% (n=24) in both the EC and LF. The differences were significant between women at labor vs. pregnant women (***, P < 0.001; for LF samples) or nonpregnant women (**, P<0.01) (Tukey’s Multiple Comparison Test). The C3 activation percentages in samples taken during pregnancy were significantly higher in the LF (79 ± 20%; n=49) than in the EC (57 ± 30%; n=49); (***, P < 0.001). No difference between LF and EC in samples taken at labor were seen.

The average C3 activation level was 72 ± 13% (mean ± SD, n=23) in the non-pregnant control group, 78 ± 22% (n=49) in the pregnant group and 58 ± 22% (n=24) during active labor (**Figure 4**). Differences were significant between the pregnant group and the labor group in EC samples (P<0.001), and between the non-pregnant group and the labor group (p<0.01) (**Figure 4**). The results indicate that C3 in this local environment is in a continuous state of activation. In ten additional swabs immersed directly into 10 mM EDTA for control of possible *ex vivo* C activation the percentages of cleaved C3 in the PBS/EDTA-containing buffer were 62 ± 23% (mean ± SD), and 70 ± 18% in the EC and LF samples, respectively. In the non-EDTA containing samples the respective percentages of C3 activation were 62 ± 21% s and 87 ± 22% in the EC and LF samples. This indicated that, unlike in EC samples, in the LF samples, some C activation continued still *ex vivo*.

### C3 Activation in Lateral Fornix Compared to External Cervix

In LF and EC samples from pregnant women and women in labor large amounts of C3 activation fragments indicated strong local activation of C3. A higher level of C3 activation was observed during pregnancy in LF: 79 ± 20% (n=49) in comparison to EC: 57 ± 30% (n=49) (P<0.001) (**Figure 4**). No significant difference was seen in values in samples from different locations during labor.

### Complement Activation by IgG

**Figure 5:**
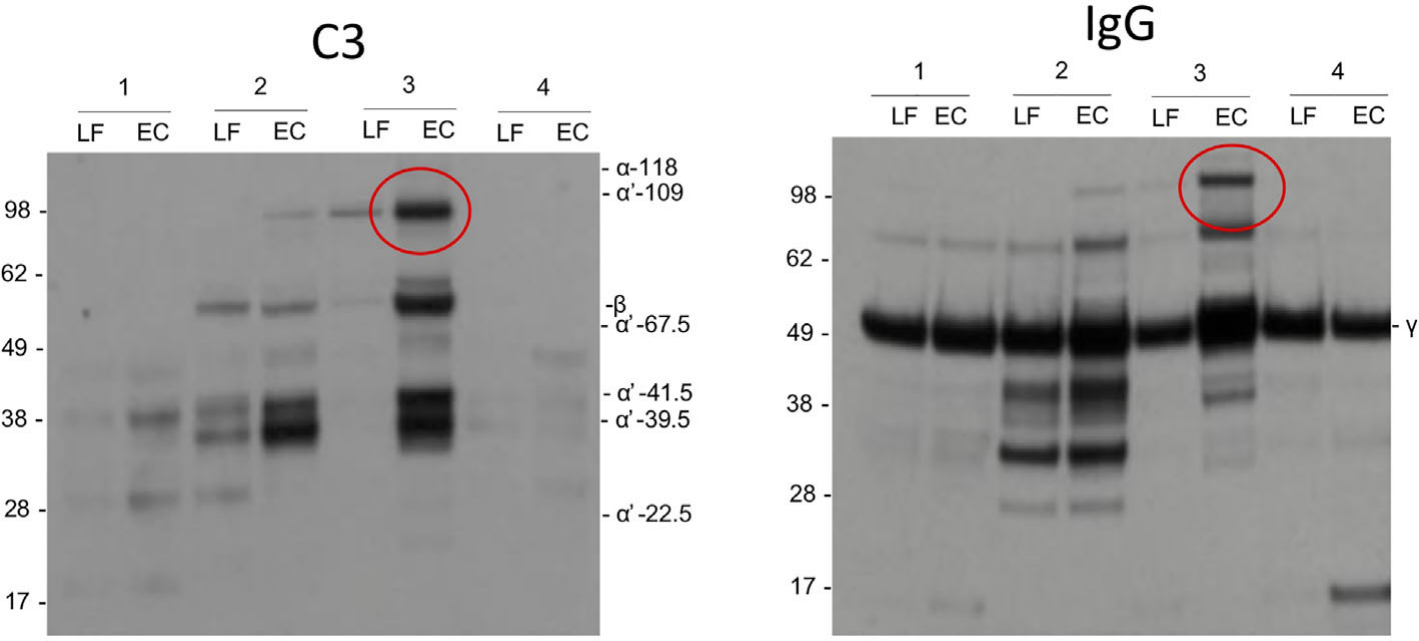
Complement activation by IgG. As evidence for complement activation by IgG we detected in some samples by Western blotting high m.w. bands (circled) that stained both for IgG heavy chain and C3. These bands may present SDS- and reduction-resistant covalent complexes between the IgG heavy chain (50 kDa) and a C3 activation fragment. The C3-IgG complexes could indicate local complex formation between IgG and C3b and thus C3 activation by the IgG antibodies.

As possible evidence for complement activation by IgG we detected in some samples by Western blotting high molecular weight bands that stained both for the IgG heavy chain and C3 (**Figure 5**). These bands could present SDS- and reduction-resistant covalent complexes between the IgG heavy chain (50 kDa) and C3 activation fragments. The C3-IgG complexes were observed in 10% of the pregnant women, in 33% of women at delivery and in 12% of control women. These complexes could indicate local complex formation between IgG and C3b and thus C3 activation by the IgG antibodies. No similar bands were detected for IgA.

## Discussion

We observed robust local complement activation in the cervicovaginal milieu. C3 activation was seen in women in all the three different groups: when not pregnant, during pregnancy and during labor. High amounts of C3 cleavage products suggest that the complement system at this site is under continuous state of activation. During labor the complement C3 amounts in samples from the cervicovaginal space appeared as the highest, but the activation percentages were the lowest. IgG was more abundantly present in the reproductive secretions during pregnancy and labor in comparison to the nonpregnant state. In contrast, IgA levels did not differ between the study groups.

Unlike usually assumed for mucosal surfaces, IgG is detected in the cervicovaginal area in addition to IgA (35). We observed higher levels of IgG than IgA in samples from women, who were pregnant or in labor. The opposite, higher levels of mucosal IgA than IgG, was true for nonpregnant women. In body fluids containing IgG and complement, a potential for immune complex formation and complement activation exists. IgG and generation of immune complexes are needed for antibacterial and antiviral defense (36). When comparing the Western blots of C3 activation fragments and IgG from the same samples, in some samples, similar high molecular weight bands (>100 kDa) were observed (**Figure 5**) suggesting the presence of covalent complexes between the IgG heavy chain and one or more C3 fragments that remain covalently bound on structures that activated complement. The C3-IgG complexes were more commonly present in samples taken during pregnancy (33%) than in the other samples (10% and 12%). This is a further indication that local complement activation has taken place. It also suggests that local IgG-containing immune complexes could be one of the complement-activating factors. Apart from the obvious factors from the local microbial flora, the nature of local antigens and the relevance of the immune complexes during pregnancy and delivery are unknown. No similar bands were observed for IgA, which is compatible with the fact that IgA does not activate complement.

In recent years, understanding the role of sterile inflammation during pregnancy and labor has increased significantly. Although the complement system has traditionally and evolutionarily been known as part of the host defense against microbes and in causing inflammation, C also maintains homeostasis by controlling the elimination nonviable tissue components (37–39). Previous studies have suggested that the complement system is involved in pregnancy and parturition, participating in tissue remodeling, recognizing injured cells and enhancing phagocytosis (10, 21, 40). C3 is abundantly produced by the uterus, and levels of C3 increase in blood towards the end of pregnancy (26). Also, additional synthesis may occur in the local mucosa (41). Higher amounts, but a lower level, of C3 activation in the cervicovaginal interphase during labor may be related to the dilation and effacement of the cervix, enabling migration of C3 from plasma. On the basis of our results we cannot say to which extent C3 is locally produced and how much is diffusing from blood. This likely depends on the situation (e.g. more C3 from blood during delivery) and varies from patient to patient. Blood-contaminated samples (visible red color) were excluded from the analyses. By measuring absorbances at 539 nm we observed that the highest level of blood contamination in the tested samples was 3.8%. Thus, not all C3 in the samples can originate from contaminating blood.

Our results indicated that complement activation continued to some extent in the LF samples *ex vivo*, because a difference was seen between samples taken to EDTA or buffer only. In both situations (presence or absence of EDTA) the C3 activation level was higher in the LF samples. A similar difference (stronger activation in LF samples; section *Local C3 Activation in Non-Pregnant and Pregnant Women and During Labor*) was seen in pregnant women, who were not in labor, but not in women at labor. Thus, although we observed some additional C3 activation in LF samples *ex vivo*, it did not influence the general conclusions of the study.

The higher activation percentage of C3 in the LF during pregnancy could result from differences in mucosal secretions and microbiome environments that may affect C3 activation. The lack of difference in C3 activation between the two sites during labor might be explained by the changed anatomical and physiological conditions, like in the fluid and blood inflow during cervical effacement. The lower level of C3 activation during labor suggests that the C system is in this situation better controlled than other times at this local site. This could also explain the lack of difference between EC and LF samples during labor. Whether the better control of C activation in the cervicovaginal space during delivery is due to greater amounts of complement regulating proteins, possibly diffusing from circulation, remains to be studied.

Knowledge about the level of local activation and regulation of the complement system during the physiological inflammation process related to delivery is scarce. In general, the activation level of C3 in the cervicovaginal samples was exceptionally high. As examples, an earlier study on local complement activation in otitis media in children reported an up to 40% level of C3 activation (34) and in dense deposit disease a nearly 100% level of C3 activation in blood plasma could be reached (42). Like in the middle ear space in otitis media, also in vagina many potential factors could contribute to C activation and C3 cleavage. They include microbes, sperm cells, damaged host cells, migrating leukocytes and proteolytic enzymes, like plasmin or matrix metalloproteinases. The cervicovaginal area is thus a true interphase, where a homeostatic balance must persist. The lower genital tract epithelium consists of multiple cell layers of stratified squamous epithelial cells that lack tight junctions. Therefore, it can allow the movement of small molecules, including complement components, through the cell layers. The high level of estrogen at the end of pregnancy increases estradiol receptor expression in cells of the reproductive tract (epithelial cells, macrophages, stromal cells, and lymphocytes). This affects the lower genital tract immunity and promotes complement C3 synthesis (43). What we observed, could thus partially be due to locally produced C3. While complement activation likely has a beneficial function, it may also be harmful. Maternal systemic complement overactivity may result in fetal damage, coagulation disorders, or excessive bleeding during delivery and postpartum. Therefore, complement needs to be carefully controlled. Complement activation can be regulated by local inhibitors or by those coming from circulation. The regulation of complement in this local environment merits more studies.

Quantification of IgG suggested that like for C3, IgG was more abundant in the samples from women in labor than in the other groups. Previous studies suggest that most mucosal IgG originates from blood plasma (7). Our results suggest that IgG is naturally present in the cervicovaginal mucosa. Part of it could derive from the local immune tissue. We have previously demonstrated the presence and activation of local lymphoid tissue including B cells in the vulvovaginal area (44). The presence and activities of IgG in promoting both complement activation and opsonophagocytosis can protect the cervicovaginal space from infections. However, IgG may also make this region vulnerable to inflammation, e.g. by activation of complement and leukocytes, which can generate reactive oxygen metabolites and other mediators of inflammation. Apparently, the balance between these two phenomena varies according to the different physiological challenges, including pregnancy and delivery. It may also depend on the expression of cellular receptors for both activated complement components, like CR1, CR3, C5aR type 1 and for IgG, i.e. the Fc receptors. The different types of Fc receptors can bind different subclasses of IgG and be linked either to activating or inhibiting submembranous domains. The net effects of receptor interactions could thus range from proinflammatory activation to more homeostatic clearance responses. During and after delivery, the need for repair processes is naturally very high. Activation of C3 to C3b (for CR1), and especially for iC3b (for CR3) thus probably plays an essential role in promoting clearance of damaged tissue components and healing at the end. In addition, antigen-bound C3dg and C3d could, *via* their receptor CR2 (CD21), favor activation of B cells and antibody synthesis in the local immune tissue. C5a in turn would promote inflammation through its phlogistic activities via C5aR1.

Hereby we have demonstrated a key activity, activation of complement C3, in the local cervicovaginal immune system. What are the beneficial functions and what are the potential risks need to be analyzed in further studies. Also, the present study did not address the complement inhibitors, which could operate to avoid a full-blown complement activation before the baby enters the birth canal. The presence of local, mucosal IgG implies that this area is an immunologically intermediate region, where both mucosal IgA-based immunity and parenteral IgG-based active immunity coexist. Both may be needed for an active, yet not too excessive inflammatory immune response against potential pathogens. Whether there also is a more direct effect on successful pregnancy or delivery remains an important topic for further studies.

The main strength of the study is the unique sample material obtained from volunteers. The samples were precisely timed, localized and in all cases taken by two experienced doctors. Pregnant women were sampled in the “late term” stage, during which, for unknown reasons, the labor had not yet started. Whether delayed delivery is related in any way to complement dysfunction is not known. Multiple activities of complement in inflammation, in regulating blood flow, cell migration, and activation suggest that complement could have a role in the induction of labor.

In conclusion, our study demonstrates a robust local activation of the complement system and the presence of IgG, in addition to IgA, in the cervicovaginal interface both under normal circumstances, during pregnancy and during delivery. This indicates the presence of an active humoral immune system in this important area. The physiological and potential pathophysiological consequences of this phenomenon remain to be worked out in further studies.

## Data Availability Statement

The original contributions presented in the study are included in the article/supplementary material. Further inquiries can be directed to the corresponding authors.

## Ethics Statement

The studies involving human participants were reviewed and approved by Helsinki University Hospital’s Ethical Committee (91/13/03/03/2015). The patients/participants provided their written informed consent to participate in this study.

## Author Contributions

SL and JH-E collected samples and. performed the measurements, HJ, IL, and IK were involved in planning and supervised the work, SL, HJ, IK, and SM processed the experimental data, performed the analysis, drafted the manuscript and designed the figures. SL, HJ, IK, IL PN, and SM aided in interpreting the results and worked on the manuscript. All authors contributed to the article and approved the submitted version.

## Funding

The study was supported by the the Sigrid Jusélius Foundation (4708373 to SM, P52483 to IK), the Academy of Finland (292393 to SM, 324944 to IK) and State Funding to the Helsinki University Hospitals (TYH, VTR): TYH2017110 (PN), TYH2020401 (IK) and TYH2018313, TYH2019311 (SM).

## Conflict of Interest

The authors declare that the research was conducted in the absence of any commercial or financial relationships that could be construed as a potential conflict of interest.
